# Supplementary material for: The Construction and Exploration of a Comprehensive MicroRNA Centered Regulatory Network in Foxtail Millet (Setaria italica L.)
Source: Front Plant Sci. 2022 May 6;13:848474. doi: 10.3389/fpls.2022.848474 (PMC9121102; doi:10.3389/fpls.2022.848474)
Supplement: Supplementary file 4 [file Data_Sheet_4.zip › Sit-miRNovel4_Seita.6G092000.1_5411_TPlot.pdf]

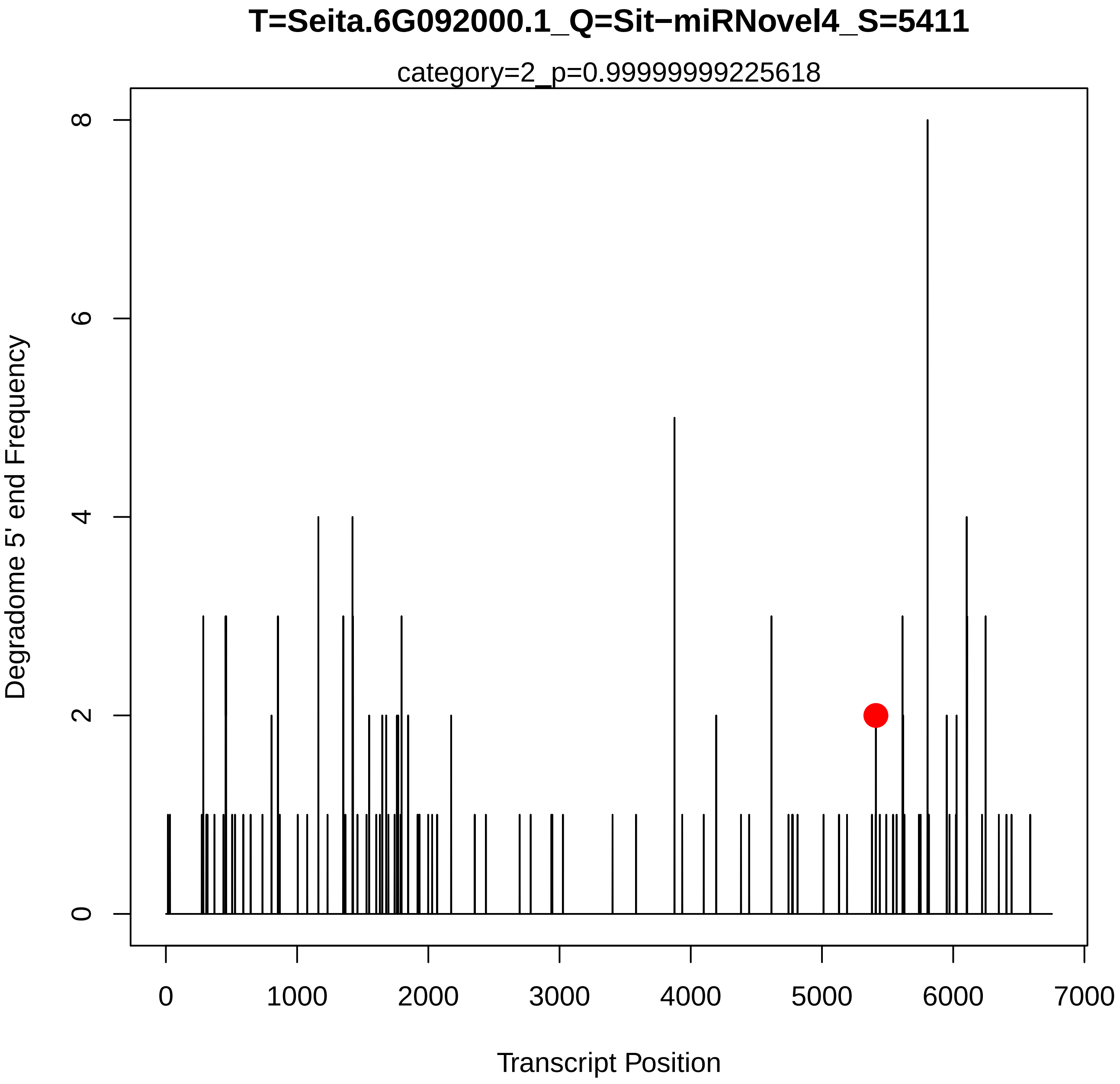

T=Seita.6G092000.1_Q=Sit−miRNovel4_S=5411
category=2_p=0.99999999225618
Degradome 5' end Frequency
0
2
4
6
8
0
1000
2000
3000
4000
5000
6000
7000
Transcript Position
